# LincRNA‐EPS inhibits caspase‐11 and NLRP3 inflammasomes in gingival fibroblasts to alleviate periodontal inflammation

**DOI:** 10.1111/cpr.13539

**Published:** 2023-09-14

**Authors:** Anni Hu, Fan Xiao, Wenjing Wu, Huilin Xu, Jiansheng Su

**Affiliations:** ^1^ Department of Prosthodontics Stomatological Hospital and Dental School of Tongji University, Shanghai Engineering Research Center of Tooth Restoration and Regeneration Shanghai China

## Abstract

To investigate the effects of long intergenic noncoding RNA‐erythroid prosurvival (lincRNA‐EPS) on periodontal inflammation mediated by inflammasomes and to explore its mechanism. Experimental periodontitis was induced in KO (*lincRNA‐EPS* ^−/−^) and WT (*lincRNA‐EPS* ^+/+^) mice to compare the periodontal bone loss and inflammation by using micro‐computed tomography, immunofluorescence staining and haematoxylin and eosin staining. The expression and activation of cysteinyl aspartate‐specific proteinase‐11 (caspase‐11) and NOD‐like receptor protein 3 (NLRP3) inflammasomes, as well as nuclear factor‐kappa B (NF‐κB) activation in mouse gingival fibroblasts (MGFs), were measured by real‐time quantitative polymerase chain reaction, Western blotting, enzyme‐linked immunosorbent and lactate dehydrogenase assays. MGFs were transfected with overexpression plasmids to assess the biological functions of lincRNA‐EPS. RNA pull‐down and immunoprecipitation experiments were performed to identify the interacting protein of lincRNA‐EPS. LincRNA‐EPS‐expressing lentivirus was locally administered to inflamed periodontal tissues to evaluate its salvage function in periodontitis. The absence of lincRNA‐EPS increased bone loss and expression of myeloperoxidase, interleukin‐1α (IL‐1α) and IL‐1β in the inflammatory periodontium. LincRNA‐EPS KO MGFs exhibited increased expression and activation of caspase‐11/NLRP3 inflammasome components than WT MGFs under lipopolysaccharide (LPS) stimulation. The expression and activation of these molecules were inhibited in lincRNA‐EPS overexpressed MGFs. Mechanistically, lincRNA‐EPS directly bound to transactive response DNA‐binding protein 43 (TDP43) in the nucleus of MGFs, and TDP43 knockdown exerted a similar inhibitory effect on NF‐κB activation and the inflammasomes as lincRNA‐EPS overexpression. Locally injecting lincRNA‐EPS‐expressing lentivirus weakened the periodontal damage. LincRNA‐EPS inhibits the LPS‐induced production and activation of caspase‐11 and NLRP3 inflammasomes by suppressing the activation of the NF‐κB signalling pathway via interacting with TDP43, thereby alleviating periodontitis.

## Graphical abstract

In the lipopolysaccharide‐induced inflammatory environment, TDP43, as a co‐activator of NF‐κB, can promote the molecular expression of caspase‐11 and NLRP3 pathways. Whereas the overexpressed lincRNA‐EPS in MGFs may competitively reduce the amount of TDP43 that co‐activated with NF‐κB through its interaction with TDP43, to weaken the transcriptional expression of caspase‐11 and NLRP3 inflammasome components, namely, NLRP3, caspase‐1, caspase‐11, IL‐1α and IL‐1β, and subsequently inhibit the activation of these inflammasomes, thereby alleviating periodontitis.

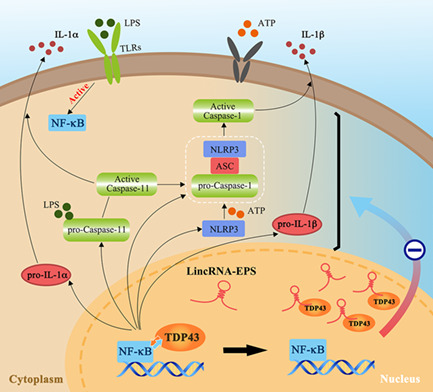

## 1 INTRODUCTION

The core feature of periodontitis is the immunoinflammatory responses of periodontal tissue to pathogens.^1^ When faced with complex environmental stimuli, cells in oral mucosal tissues will activate inflammatory signals including nuclear factor‐kappa B (NF‐κB) and inflammasome pathways to start the early innate immune defence and infection clearance.^2^, ^3^, ^4^ However, the persistent and excessive inflammatory cascades will lead to an imbalance of the anti‐inflammatory and pro‐inflammatory immune responses in the host, which not only causes mucosal inflammation, bone resorption and tooth loss but also aggravates the risk of systemic diseases (such as diabetes, neurodegenerative diseases and cardiovascular diseases^5^, ^6^, ^7^). By now, the conventional treatment schemes for periodontitis mainly rely on the mechanical disruption and removal of microbial biofilm. Although these therapies have certain effects in controlling pathogen attacks and improving clinical symptoms, they still have drawbacks of susceptibility to recurrence and do not involve host‐targeted inflammation control and immune regulation.^2^ Thus, it is important to explore new therapeutic approaches to periodontal diseases.

The inflammasome, as a hub of innate immunity, is the major complex mediating the secretion of key pro‐inflammatory factor interleukin‐1 (IL‐1).^8^, ^9^ When stimulated, canonical inflammasomes such as NOD‐like receptor protein 3 (NLRP3) and non‐canonical inflammasomes such as cysteinyl aspartate‐specific proteinase‐4/5 (caspase‐4/5) in humans or caspase‐11 in mice are activated downstream of NF‐κB signalling, leading to the release of IL‐1α and IL‐1β, along with pyroptosis.^8^, ^10^, ^11^ Compared with healthy subjects, the expression of NLRP3, NLRP6, caspase‐1 and IL‐1β in the gingival tissues of patients is upregulated to varying degrees.^12^, ^13^, ^14^ Recent studies have shown that NLRP3 and caspase‐4 inflammasomes in human periodontal ligament stem cells (PDLSCs) and gingival fibroblasts (GFs) can be activated under an inflammatory environment, resulting in pyroptosis and IL‐1β secretion, exacerbating periodontitis.^4^, ^15^ Therefore, proper control of inflammasomes may be the key element contributing to the treatment of periodontitis.

Studies have supported that lncRNAs participate in the pathogenesis of periodontitis by modulating inflammatory responses, cellular processes and osteogenesis/osteoclastogenesis.^16^, ^17^ For instance, lncRNA SNHG5 mediates periodontal inflammation by suppressing the activation of NF‐κB signalling and NLRP3 expression.^18^ The lncRNA NRON can inhibit osteoclastogenesis and alveolar bone resorption.^19^ However, the specific role of lncRNAs in modulating inflammasomes in the case of periodontitis is still poorly understood.

The long intergenic noncoding RNA‐erythroid prosurvival (lincRNA‐EPS) is reported to inhibit the expression of immune response genes (IRGs) as well as activation of NLRP3 inflammasome and to play a role in regulating inflammation in certain diseases.^20^ For instance, lincRNA‐EPS represses the HMGB1‐NF‐κB‐dependent inflammation of pancreatic macrophages and exerts a protective effect on acute pancreatitis.^21^ Moreover, it also attenuates ischaemia/reperfusion injury.^22^ According to Atianand et al., the function of lincRNA‐EPS in impeding IRGs and NLRP3 inflammasome activation in macrophages is realised through interacting with heterogeneous nuclear ribonucleoproteins L.^20^ Another member of the hnRNPs family, transactive response DNA‐binding protein 43 (TDP43), can also bind to lncRNAs to regulate inflammatory genes.^23^, ^24^ Recent literatures reported that TDP43 promotes the expression of NLRP3 and IL‐1β and the activation of NLRP3 inflammasome.^25^ Notwithstanding, the role and mechanism of lincRNA‐EPS in periodontitis especially on the aspect of inflammasome regulation, and, in addition, whether lincRNA‐EPS is associated with TDP43 have not been reported yet.

Our pilot study showed that the expression of lincRNA‐EPS was restricted in the inflamed mouse periodontal tissues, and its deficiency caused more severe periodontitis, including increased expression of IL‐1α/β, suggesting that it might be related to the inflammasomes in periodontal inflammation. Accordingly, the present study aimed to investigate whether lincRNA‐EPS functions in modulating inflammasomes in periodontal inflammation and to explore its specific mechanism to identify a potential therapeutic target for the treatment regimen of periodontal disease.

## 2 MATERIALS AND METHODS

### 2.1 Mice and ligature‐induced periodontitis

KO (*lincRNA‐EPS* ^−/−^) mice on a C57BL/6 background were generated by Shanghai Model Organisms Center, Inc. C57BL/6 mice were housed in Shanghai Engineering Research Center of Tooth Restoration and Regeneration.

**FIGURE 1:**
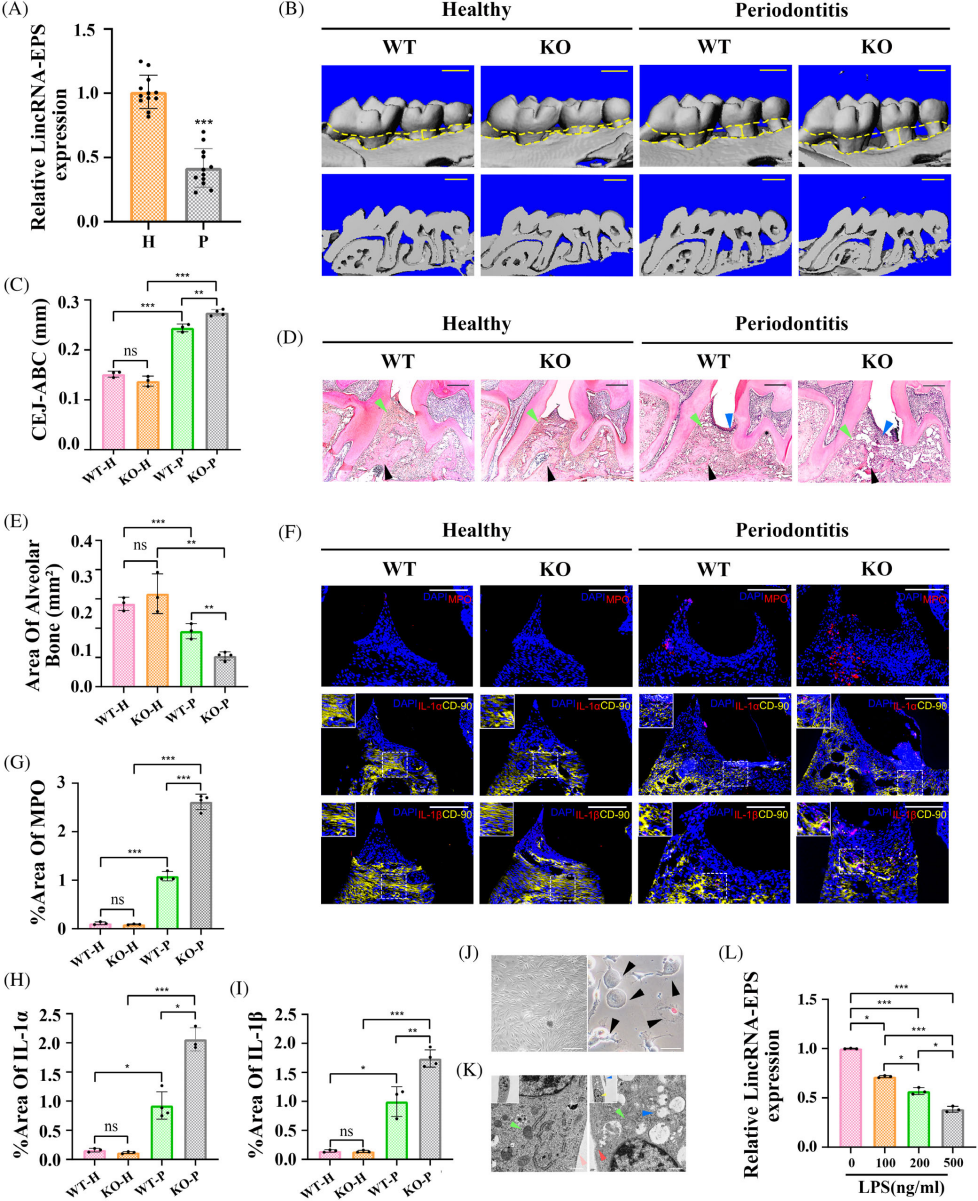
LincRNA‐EPS was downregulated in inflamed periodontal tissues and gingival fibroblasts; lincRNA‐EPS KO aggravated periodontitis. (A) Detection of lincRNA‐EPS expression in the periodontal tissues from mice with or without periodontitis (RT‐qPCR; *n* = 12). Endogenous control: β‐actin. (B) Reconstructed 3D and 2D micro‐CT images of periodontal tissues from WT and KO mice, scale bar = 1 mm. (C) The distances (mm) of cementoenamel junction to alveolar bone crest (CEJ‐ABC) of periodontal tissues from respective groups. (D) Representative haematoxylin and eosoin (H&E) staining for periodontal tissues from respective groups, scale bar = 200 μm. Alveolar bone (black arrow), periodontal ligament (green arrow), inflammatory cell infiltration (blue arrow). (E) The area of alveolar bone (mm^2^) of periodontal tissues from respective groups. (F) Representative immunofluorescence staining of MPO, IL‐1α, IL‐1β (red) and CD90 (yellow) in the periodontal tissues, the co‐localisation (orange) of IL‐1α or IL‐1β with CD90 antibodies was magnified in the square box. Nuclei were identified by staining with DAPI (blue), scale bar = 100 μm. Percentage of MPO (G), IL‐1α (H), IL‐1β (I) stained area over total area. (J) The primary mouse gingival fibroblasts before the first passage (left image), scale bar = 1000 μm. Arrowheads indicate ballooned cell membrane and PI‐positive staining (red) of pyroptotic cells (right image), scale bar = 100 μm. (K) MGFs were detected by transmission electron microscope with or without LPS + ATP stimulation, scale bar = 500 nm. Nucleus pyknosis (yellow arrow), mitochondria (green arrow), intact cell membrane (pink arrow), broken cell membrane (red arrow), vesicles (blue arrow). (L) LincRNA‐EPS expression in MGFs treated with LPS at different concentrations (0, 100, 200, 500 ng/mL) and ATP (RT‐qPCR; *n* = 3). Endogenous control: β‐actin. Data are presented as mean ± SD. *p < 0.05; **p < 0.01; ***p < 0.001. H, healthy group; KO, periodontal tissues from KO mice; ns, no significance; P, periodontitis group; WT, periodontal tissues from WT mice.

Under anaesthesia, the maxillary right second molars of 8‐week‐old male C57/BL6 mice were ligated with silk, and the opposite side was used as a healthy control. The mice were euthanized under anaesthesia on the eighth day, and periodontal tissue samples were harvested. For the real‐time quantitative polymerase chain reaction (RT‐qPCR) analysis shown in Figure 1A, 24 male C57BL/6 mice were included in the experiment (gingival tissues from 2 mice were used for each sample, *n* = 12).

Animal experiments were approved by the Ethics Review Board of the Affiliated Stomatology Hospital of Tongji University (NO. [2020]‐DW‐02) and were performed in compliance with ARRIVE guidelines.

### 2.2 Tissue specimens, haematoxylin and eosin and immunofluorescence staining

The periodontal tissues were fixed with 4% paraformaldehyde (Beyotime) for 48 h, decalcified with a 14% ethylenediaminetetraacetic acid solution (Macklin) and then embedded in paraffin for sectioning. The sections were deparaffinized and hydrated subsequently. For haematoxylin and eosin (H&E) staining, the sections were stained with H&E. For tissue immunofluorescence (IF) staining, the sections were then incubated with hyaluronidase (2 mg/mL; Sigma) for 1 h at 37°C for antigen retrieval. After blocking with 1% BSA (Beyotime), the sections were incubated overnight at 4°C with myeloperoxidase (MPO), IL‐1α, IL‐1β and CD90 primary antibodies respectively. Afterwards, the sections were incubated with corresponding secondary antibodies for 1 h and then counterstained with DAPI (Beyotime). Images were captured with a high‐resolution digital microscope (Nikon). The percentage of the positively stained area of IF staining was measured by ImageJ software.

### 2.3 Cell treatments

Cells were maintained in complete Dulbecco's modified Eagle's medium (DMEM; HyClone) overnight. The medium was next replaced with the serum‐free medium on the next morning. Mouse gingival fibroblasts (MGFs) were divided into three treatment groups: mock group (untreated control), lipopolysaccharide (LPS) group (cells were primed with *Escherichia coli* LPS [Sigma‐Aldrich] at 100 ng/mL in DMEM) for 6 h and LPS + ATP group (cells were primed with LPS for 6 h and then treated with ATP at 5 mM [BBI Life Science] during the last 2 h).

### 2.4 Real‐time quantitative polymerase chain reaction

Total RNA was extracted from gingival tissues or cells with TRIzol reagent (Takara). One thousand nanograms of RNA were used to synthesise cDNAs with RT Master Mix (Takara). qPCR was performed using SYBR Green Reagent (Yeasen) with specific primers and LightCycler 96 (Roche) (Table 1). The cycle threshold (*C*_t_) was recorded, and gene expression was determined with 2^−ΔΔ*C*t^ method. U6, GAPDH and β‐actin were used as endogenous controls for different tests.

**TABLE 1:** Primers for RT‐qPCR.

Gene	Primers
LincRNA‐EPS	Forward: 5′‐GAGCCAGACATCCACCCAG‐3′
	Reverse: 5′‐TGGCTGTGTTCTTGTGGGTT‐3′
LincRNA‐EPS1	Forward: 5′‐ATGTGTATTAGAGTTTTGCCT‐3′
	Reverse: 5′‐TCTTTTCAAGCCCATATGTGA‐3′
β‐Actin	Forward: 5′‐GGCTGTATTCCCCTCCATCG‐3′
	Reverse: 5′‐CCAGTTGGTAACAATGCCAT‐3′
U6	Forward: 5′‐CTCGCTTCGGCAGCACA‐3′
	Reverse: 5′‐AACGCTTCACGAATTTGCGT‐3′
GAPDH	Forward: 5′‐AGGTCGGTGTGAACGGATTTG‐3′
	Reverse: 5′‐TGTAGACCATGTAGTTGAGGTCA‐3′
NLRP3	Forward: 5′‐ATTACCCGCCCGAGAAAGG‐3′
	Reverse: 5′‐TCGCAGCAAAGATCCACACAG‐3′
Caspase‐1	Forward: 5′‐ACAAGGCACGGGACCTATG‐3′
	Reverse: 5′‐TCCCAGTCAGTCCTGGAAATG‐3′
Caspase‐11	Forward: 5′‐AGAAGTCCTTACGGAGTACC‐3′
	Reverse: 5′‐TGGTGTTCTGAGAGTGCAGC‐3′
IL‐1α	Forward: 5′‐ACGTCAAGCAACGGGAAGAT‐3′
	Reverse: 5′‐AAGGTGCTGATCTGGGTTGG‐3′
IL‐1β	Forward: 5′‐GACTCCTTAGTCCTCGGCCA‐3′
	Reverse: 5′‐GTGCTGCCTAATGTCCCCTT‐3′
TDP43	Forward: 5′‐CCTTTGCAGATGATAAGGTTGCC‐3′
	Reverse: 5′‐TGTGCAGCGTGATGACGAA‐3′
RelA	Forward: 5′‐AGGCTTCTGGGCCTTATGTG‐3′
	Reverse: 5′‐TGCTTCTCTCGCCAGGAATAC‐3′
IL‐6	Forward: 5′‐TAGTCCTTCCTACCCCAATTTCC‐3′
	Reverse: 5′‐TTGGTCCTTAGCCACTCCTTC‐3′
COX‐2	Forward: 5′‐TTCAACACACTCTATCACTGGC‐3′
	Reverse: 5′‐AGAAGCGTTTGCGGTACTCAT‐3′
TNF‐α	Forward: 5′‐TTCAACACACTCTATCACTGGC‐3′
	Reverse: 5′‐AGAAGCGTTTGCGGTACTCAT‐3′

*Note*: Sequence of lincRNA‐EPS1 showed the most significant enrichment in the RT‐qPCR assay after RIP.

Abbreviations: RIP, RNA immunoprecipitation; RT‐qPCR, real‐time quantitative PCR.

### 2.5 In vitro gene overexpression and knockdown

The full‐length lincRNA‐EPS was cloned into pZW1‐snoVetor (Addgene) and confirmed by DNA sequencing to obtain lincRNA‐EPS plasmids. TDP43 siRNAs were purchased from RiboBio Biotechnology. Cells were cultured until 60% confluence was achieved, then transfected with the plasmids or siRNAs using Lipofectamine 3000 (Thermo Fisher Scientific) and cultured for 24 h before stimulation. The sequences are listed in Table S1.

### 2.6 Fluorescence in situ hybridization followed by IF staining

The fluorescent probes against U6 snRNA, 18S rRNA and lincRNA‐EPS were synthesised and designed by RiboBio (RiboBio Biotechnology, Guangzhou, China). MGFs were maintained on slices with indicated treatments. A fluorescence in situ hybridization (FISH) experiment was performed using the Fluorescent in situ Hybridization Kit (RiboBio) as directed by the manufacturer. Then, the fluorescence‐labelled cells were incubated with a rabbit anti‐TDP43 antibody and DAPI as described above. A laser confocal microscope system (Nikon) was used to capture the images.

### 2.7 Biotin‐labelled RNA pull‐down and mass spectrometry

After linearization of plasmids, in vitro T7 RNA polymerase transcription (Large Scale RNA Production System‐T7; Promega) and Biotin RNA Labeling (RNA 3′ End Desthiobiotinylation Kit; Thermo Fisher Scientific) were used to synthesise transcripts of lincRNA‐EPS full‐length and mutant fragments. The primers are presented in Table S2. Meanwhile, protein lysates from LPS + ATP‐treated NIH3T3 cells were prepared. Then, the biotin RNAs were incubated with the lysate overnight, and a pull‐down assay was implemented in accordance with the instructions of the Pierce Magnetic RNA–Protein Pull‐Down Kit (Thermo Fisher Scientific). Finally, the retrieved proteins were detected by mass spectrometry (MS) at Shanghai Dian Xi Bio Co. Ltd.

### 2.8 RNA immunoprecipitation assay

LincRNA‐EPS RNA immunoprecipitation (RIP) assay was conducted by using Magna RIP RNA‐binding Protein Immunoprecipitation Kit (Merck Millipore). In brief, magnetic beads were incubated with the TDP43 antibody and the negative control immunoglobulin G (IgG) antibody. NIH3T3 cells were treated with LPS + ATP, lysed with lysis buffer and then incubated overnight with antibody‐conjugated magnetic beads. After immunoprecipitation with the antibodies, the RNA samples were purified with phenol: chloroform:isoamyl alcohol and subsequently subjected to RT‐qPCR for the assessment of enrichment of lincRNA‐EPS with TDP43 antibody as compared to IgG.

### 2.9 Statistical analysis

The statistical analysis was performed with the statistical software R version 3.5.3 and data are presented as means ± standard deviation (SD) using GraphPad Prism version 8.02 (GraphPad Software). Data were evaluated with *t*‐tests or Mann–Whitney *U* test between two groups. For the comparisons among groups, analysis of variance was used when the variance was homogeneous or Kruskal–Wallis *H* test if the variance was not homogeneous. A value of *p* < 0.05 was considered statistically significant.

The following methods are described in the Supporting Information Materials: antibodies, micro‐computed tomography (micro‐CT) analysis, cell culture, transmission electron microscopy (TEM), propidium iodide (PI) staining, protein extraction and Western blotting, enzyme‐linked immunosorbent assay (ELISA), lactate dehydrogenase (LDH) assay, cytosolic and nuclear fractionation and RNA/protein isolation, lincRNA‐EPS lentiviral expression vector construction and topical injection.

## 3 RESULTS

### 3.1 LincRNA‐EPS was downregulated in inflamed periodontal tissues, and lincRNA‐EPS KO aggravated periodontitis

A periodontitis model was established in mice to investigate the potential effect of lincRNA‐EPS on periodontal inflammation. As shown in RT‐qPCR, the expression of lincRNA‐EPS significantly decreased in the gingiva from periodontitis tissues as compared to healthy controls (Figure 1A). When comparing periodontitis tissues from WT and KO (*lincRNA‐EPS* ^−/−^) mice, micro‐CT analysis showed that the silk ligation increased the distance from the cementoenamel junction to the alveolar bone crest (CEJ‐ABC) of the maxillary second molar, while reduced the area of alveolar bone around the second molar. Among the periodontitis groups, the CEJ‐ABC distance in the KO group was significantly greater than that in the WT group, while the area of the alveolar bone was significantly decreased (Figure 1B,C,E). H&E staining also demonstrated that lincRNA‐EPS KO did not affect the healthy periodontium, but under periodontitis conditions, KO tissues showed more obvious bone resorption, periodontal ligament destruction and inflammatory cell infiltration than WT tissues (Figure 1D). According to IF staining, we observed that in inflammatory tissues, the expression of MPO (a specific marker of polymorphonuclear neutrophils^2^, ^26^), IL‐1α and IL‐1β were higher in the KO group than that in the WT group, where MPO marked a large accumulation of inflammatory cells. In addition, IL‐1α and IL‐1β were both co‐localised with CD90 (one of the markers of PDLSCs and GFs^15^, ^27^) in the inflamed periodontium from WT and KO group, while more colocalized points were observed in the KO group than in the WT group (Figure 1F–I). This suggests the expression of lincRNA‐EPS was restrained in inflamed periodontal tissues, and it may exert a negative effect on the inflammatory cell infiltration and IL‐1α/β expression (especially in PDLSCs and MGFs) in the inflamed periodontium.

### 3.2 LincRNA‐EPS was observable in MGFs and reduced by LPS

GFs, the main resident cells of gingival mucosa, are one of the forefront cells of periodontium responding to infection.^28^ Whether lincRNA‐EPS involves in inflammasome‐mediated inflammation in MGFs was further investigated. First, LPS and ATP were used to stimulate primary MGFs and activate NLRP3 inflammasomes. The treated MGFs exhibited microscopic features of pyroptosis: ballooning of cell membrane and positive PI staining (Figure 1J).^10^, ^15^, ^29^, ^30^ The TEM images showed obvious morphologic hallmarks of pyroptotic MGFs (nucleus pyknosis, numerous intracellular vesicles, membrane rupture^13^, ^31^) (Figure 1K). In addition, lincRNA‐EPS was detectable in MGFs. Its expression was significantly downregulated after LPS + ATP treatment with the increase of LPS concentration (Figure 1L), suggesting that the lincRNA‐EPS expression was inhibited by LPS in the presence of inflammation.

### 3.3 LincRNA‐EPS KO promoted production and activation of caspase‐11 and NLRP3 inflammasomes

**FIGURE 2:**
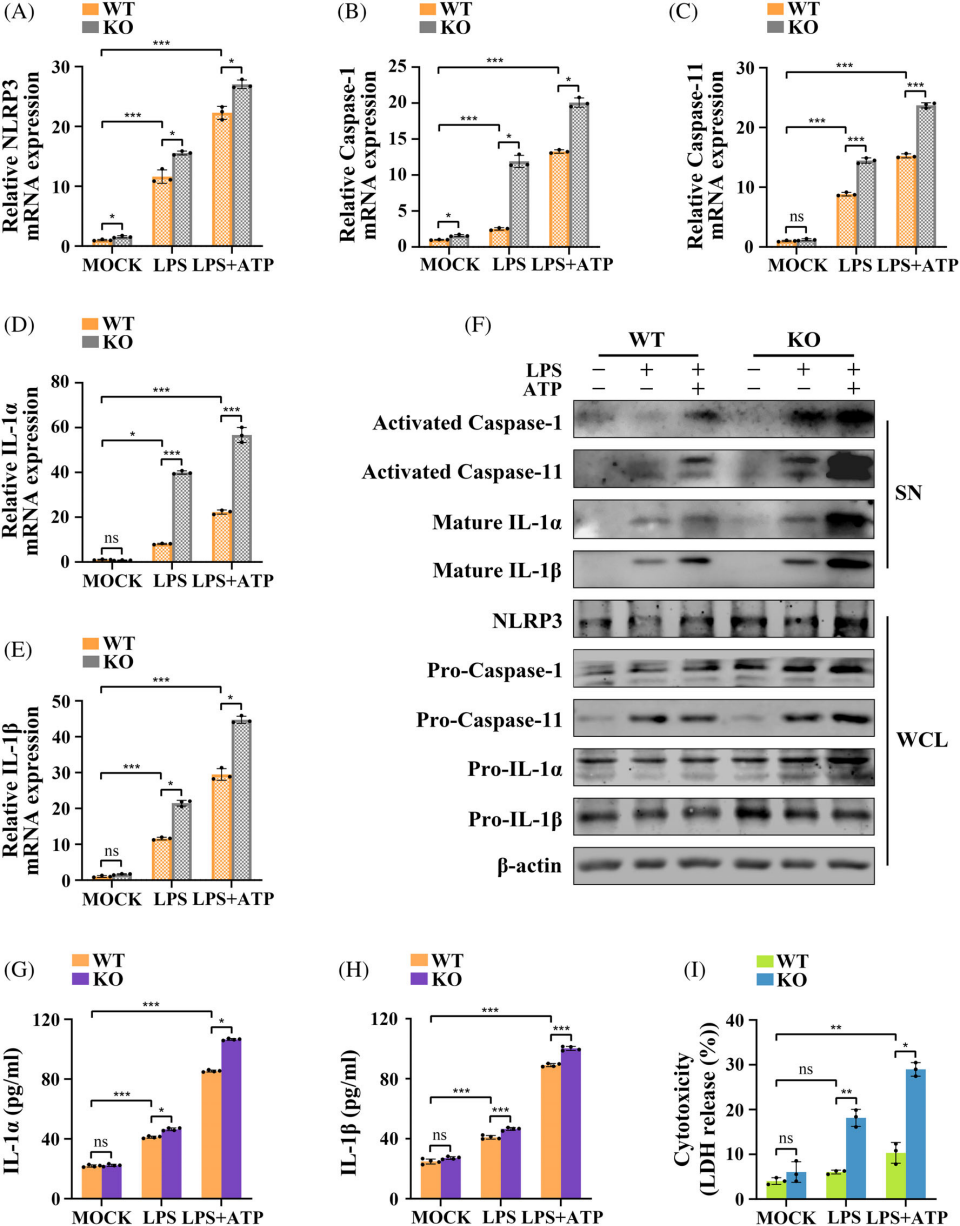
LincRNA‐EPS KO promoted the production and activation of caspase‐11 and NLRP3 inflammasomes. (A–I) MGFs from WT and KO mice were untreated, treated with LPS alone or treated with LPS + ATP. (A–E) mRNA expressions of NLRP3, caspase‐1, caspase‐11, IL‐1α and IL‐1β (RT‐qPCR; *n* = 3). Endogenous control: β‐actin. (F) The whole cell lysate (WCL) and supernatant (SN) fractions were detected by Western blotting with antibodies against indicated proteins: NLRP3, pro‐caspase‐1, pro‐caspase‐11, pro‐IL‐1α and pro‐IL‐1β in the WCL and activated caspase‐1, activated caspase‐11, mature IL‐1α and mature IL‐1β in the SN. Loading control: β‐actin. The experiments were repeated three times. (G, H) Cell supernatants were assayed for mouse IL‐1α and IL‐1β by ELISA (*n* = 4). (I) Cytotoxicity was assessed by LDH release as a percentage (%) of total LDH release (*n* = 3). (A–E, G–I) Data are presented as mean ± SD. *p < 0.05; **p < 0.01; ***p < 0.00. KO, MGFs from KO mice; LPS, treated with LPS alone; LPS + ATP, treated with LPS and ATP; MOCK, untreated without any stimulation; ns, no significance; SN, supernatant; WCL, whole cell lysate; WT, MGFs from WT mice.

MGFs from WT and lincRNA‐EPS KO mice were stimulated with LPS (with or without ATP) under the same conditions (consistent cell density and growth state) to determine whether non‐canonical inflammasome activation occurs in MGFs and whether lincRNA‐EPS exerts a regulatory effect on inflammasomes was explored. RT‐qPCR showed that mRNA levels of NLRP3, caspase‐1, caspase‐11, IL‐1α and IL‐1β were noticeably upregulated after treatment and they were the highest after LPS + ATP treatment. Notably, all the mRNA expressions of the above molecules were significantly higher in the KO group than in the WT group following LPS and LPS + ATP treatment (Figure 2A–E).

Western blotting showed the protein expressions of NLRP3, pro‐caspase‐1, pro‐caspase‐11, pro‐IL‐1α and pro‐IL‐1β in the KO MGFs were markedly higher than in the WT MGFs following LPS or LPS + ATP treatment. Notably, the end products of inflammasome activation (activated caspase‐1, activated caspase‐11, mature IL‐1α and mature IL‐1β) were undetectable in the resting cell supernatant. Upon LPS stimulation alone, the activated caspase‐11, mature IL‐1α and mature IL‐1β were detectable in small amounts in the WT MGFs, and the levels of all four activated proteins significantly increased following LPS + ATP treatment. Moreover, the expressions of all the activated end products in the KO MGFs were significantly higher than in the corresponding WT MGFs (Figures 2F and S1A–C). ELISA also showed a similar trend: the concentrations of IL‐1α and IL‐1β gradually increased after LPS and LPS + ATP treatment. More importantly, the increases in the concentrations of IL‐1α and IL‐1β in the KO group were more evident than in the WT group (Figure 2G,H).

A specific indicator of pyroptosis is LDH release in the supernatant.^11^ Our results showed lincRNA‐EPS KO had no effect on resting MGFs, but it remarkably increased LDH release in case of inflammation (Figure 2I).

### 3.4 LincRNA‐EPS overexpression inhibited the production and activation of caspase‐11 and NLRP3 inflammasomes

**FIGURE 3:**
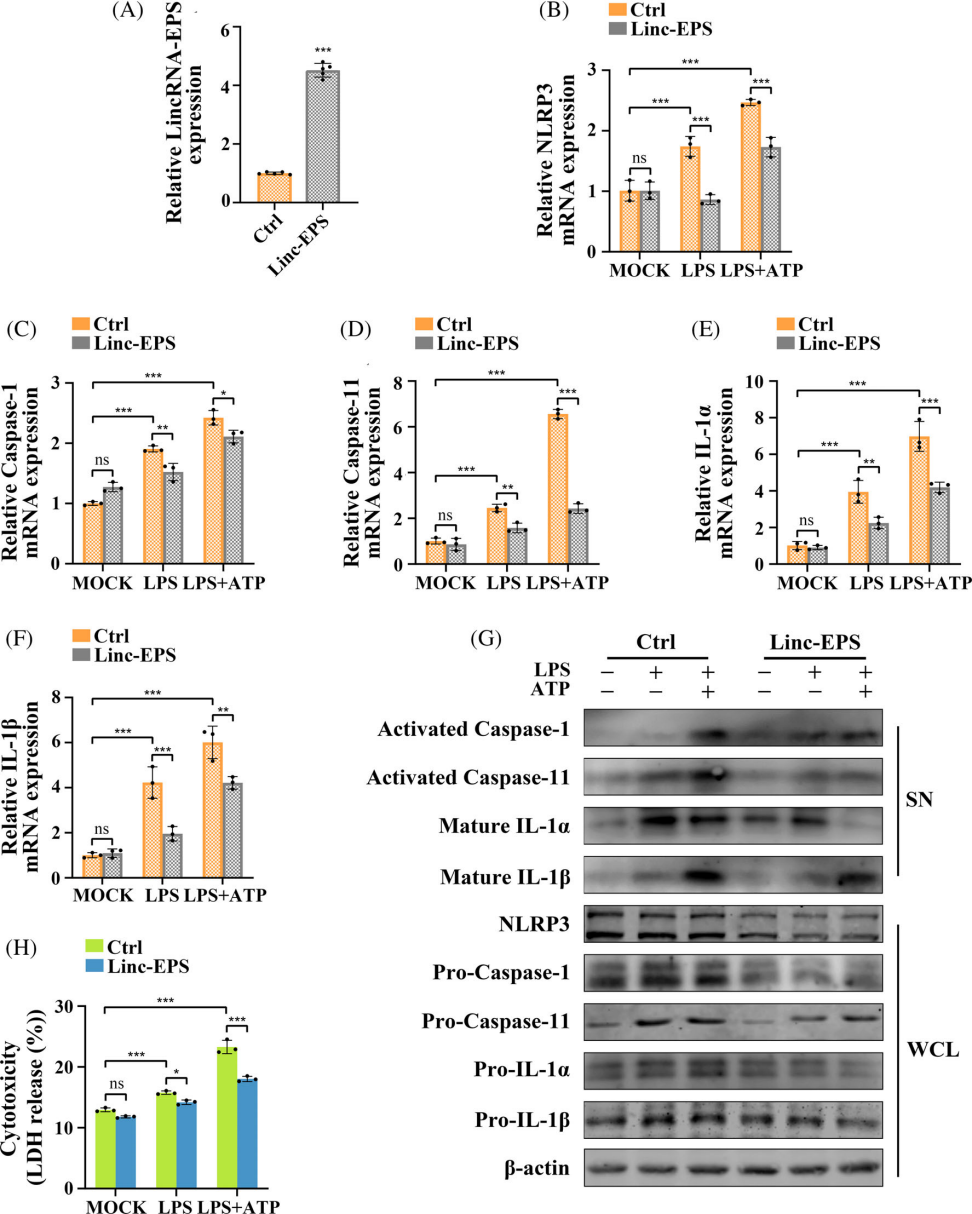
LincRNA‐EPS overexpression inhibited the production and activation of caspase‐11 and NLRP3 inflammasomes. (A) Overexpression of lincRNA‐EPS detected by RT‐qPCR (*n* = 5). Endogenous control: β‐actin. (B–H) MGFs overexpressing lincRNA‐EPS and WT negative control were untreated, treated with LPS alone or treated with LPS + ATP. (B–F) mRNA expressions of NLRP3, caspase‐1, caspase‐11, IL‐1α and IL‐1β levels (RT‐qPCR; *n* = 3). Endogenous control: β‐actin. (G) Protein expressions of indicated molecules were detected by Western blotting. Loading control: β‐actin. The experiments were repeated three times. (H) Cytotoxicity was assessed by LDH release as a percentage (%) of total LDH release (*n* = 3). (A–F, H) Data are presented as mean ± SD. *p < 0.05; **p < 0.01; ***p < 0.001. Ctrl, MGFs transfected with negative control plasmids; linc‐EPS, MGFs transfected with lincRNA‐EPS overexpression plasmids; LPS, treated with LPS alone; LPS + ATP, treated with LPS and ATP; MOCK, untreated without any stimulation; ns, no significance; SN, supernatant; WCL, whole cell lysate.

LincRNA‐EPS plasmids were transfected into WT MGFs to induce lincRNA‐EPS overexpression. RT‐qPCR revealed significantly lower mRNA expressions of NLRP3, caspase‐1, caspase‐11, IL‐1α and IL‐1β in the linc‐EPS (lincRNA‐EPS overexpression) group than in the control group after LPS or LPS + ATP treatment, but no difference was observed in resting cells (Figure 3A–F). Similarly, the protein expressions of NLRP3, pro‐caspase‐1, pro‐caspase‐11, pro‐IL‐1α and pro‐IL‐1β reduced after lincRNA‐EPS overexpression in case of inflammation. Interestingly, lincRNA‐EPS overexpression not only decreased the expressions of activated caspase‐11 and mature IL‐1α and slightly decreased the expression of mature IL‐1β following LPS treatment, but also significantly reduced the production of all four activated end products after LPS + ATP treatment (Figure 3G). Correspondingly, LDH release in the linc‐EPS group markedly decreased as compared to the control group following LPS treatment. Meanwhile, this reduction was more obvious after LPS + ATP treatment (Figure 3H). Taken together, the in vitro experiments indicate that LPS induces the production and activation of caspase‐11 and NLRP3 inflammasome in MGFs with or without ATP co‐stimulation. Most importantly, lincRNA‐EPS KO increases the expressions and activation of caspase‐11 and NLRP3 inflammasome components, while lincRNA‐EPS overexpression inhibits them.

### 3.5 LincRNA‐EPS bound to TDP43 in LPS‐induced MGFs

**FIGURE 4:**
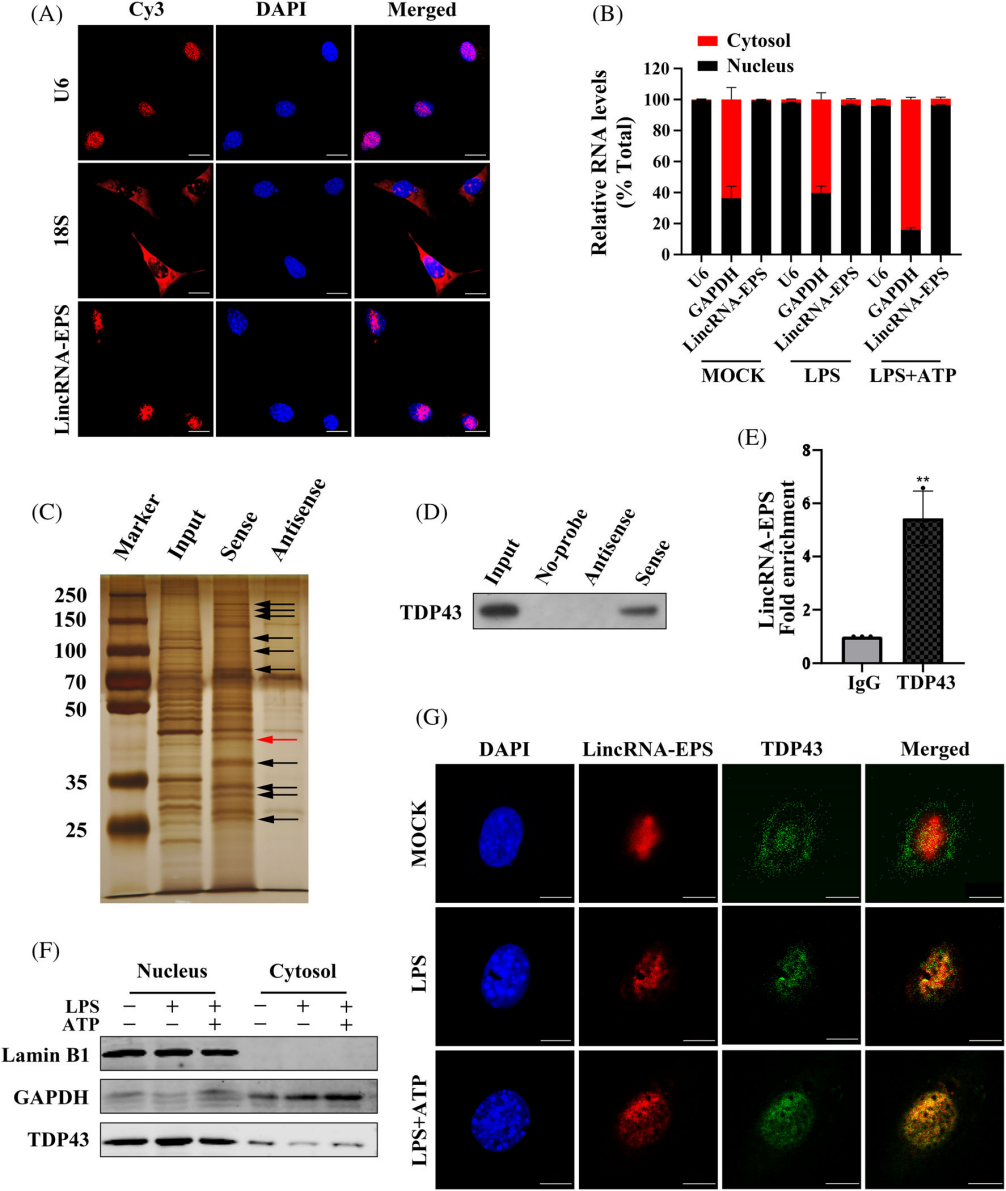
LincRNA‐EPS bound to TDP43 in an inflammatory environment. (A) RNA FISH for Cy3‐labelled 18S, U6 and lincRNA‐EPS (red). Nuclei were stained with DAPI (blue), scale bar = 20 μm. The experiments were repeated three times. (B) Nuclear and cytoplasmic RNAs in the MGFs with no treatment, LPS stimulation, or LPS + ATP stimulation (RT‐qPCR). The experiments were repeated three times (*n* = 3), and data are presented as mean ± SD. GAPDH: cytoplasmic control; U6: nuclear control. % Total: the percentage (%) of RNAs in total RNA (nuclear + cytoplasmic RNAs). (C) Silver‐stained SDS‐PAGE of proteins purified from RNA pull‐down experiments using biotin‐labelled lincRNA‐EPS sense or antisense RNA. Differentially pulled‐down protein bands are marked with arrows, and red arrow shows band at about 40 kDa. Molecular weights are annotated on the marker in kDa. (D) Western blotting confirmed the lincRNA‐EPS and TDP43 interaction using products from RNA pull‐down. (E) RIP followed by RT‐qPCR analysis of copurified RNAs in the LPS + ATP‐treated NIH3T3 cells (*n* = 3). Data are presented as mean ± SD. **p < 0.01. (F) TDP43 expression in the nuclear and cytoplasmic fractions of MGFs with no treatment, LPS stimulation, or LPS + ATP stimulation. The experiments were repeated three times. GAPDH: cytoplasmic control; Lamin B1: nuclear control. (G) LincRNA‐EPS (red) and TDP43 (green) co‐localised (yellow) in LPS‐ and LPS + ATP‐stimulated MGFs (RNA FISH and IF staining). The nuclei were identified by DAPI staining (blue), scale bar = 10 μm. The experiments were repeated three times.

The subcellular localization of lincRNA‐EPS should be first confirmed, which is helpful for the investigation of the mechanism underlying its inhibitory effect on inflammasomes. FISH and nucleocytoplasmic isolation RT‐qPCR showed that lincRNA‐EPS was mainly localised in the nucleus of resting or LPS/ATP‐stimulated MGFs (Figure 4A,B).

Then, an RNA pull‐down assay was performed in LPS + ATP‐treated NIH3T3 cells, aiming to identify the lincRNA‐EPS‐binding proteins. Silver staining showed some strong bands in the sense lane but not the antisense lane. Following MS, TDP43 attracted our attention the most (Figure 4C,D). Then, RIP was performed and results showed that lincRNA‐EPS enrichment by TDP43 antibody was markedly higher than that induced by IgG (Figure 4E), indicating that lincRNA‐EPS is able to bind to TDP43 upon LPS + ATP treatment.

Further investigation revealed that TDP43 was mainly located in the nucleus of resting MGFs, with a small amount detectable in the cytoplasm. After treatment, the cytoplasmic TDP43 decreased and it was located primarily in the nucleus (Figure 4F). At the same time, lincRNA‐EPS was mainly located in the nucleus of MGFs in both resting and inflammatory environments (Figure 4B). FISH combined with IF showed that lincRNA‐EPS had no obvious association with TDP43 in untreated MGFs, but clear co‐localisation of lincRNA‐EPS and TDP43 was observed in the nucleus after LPS or LPS + ATP treatment (Figure 4G). These suggest that the regulation of LPS‐induced inflammasomes by lincRNA‐EPS depends on its interaction with TDP43 in the nucleus of MGFs.

### 3.6 TDP43 knockdown suppressed expression of inflammasome components

**FIGURE 5:**
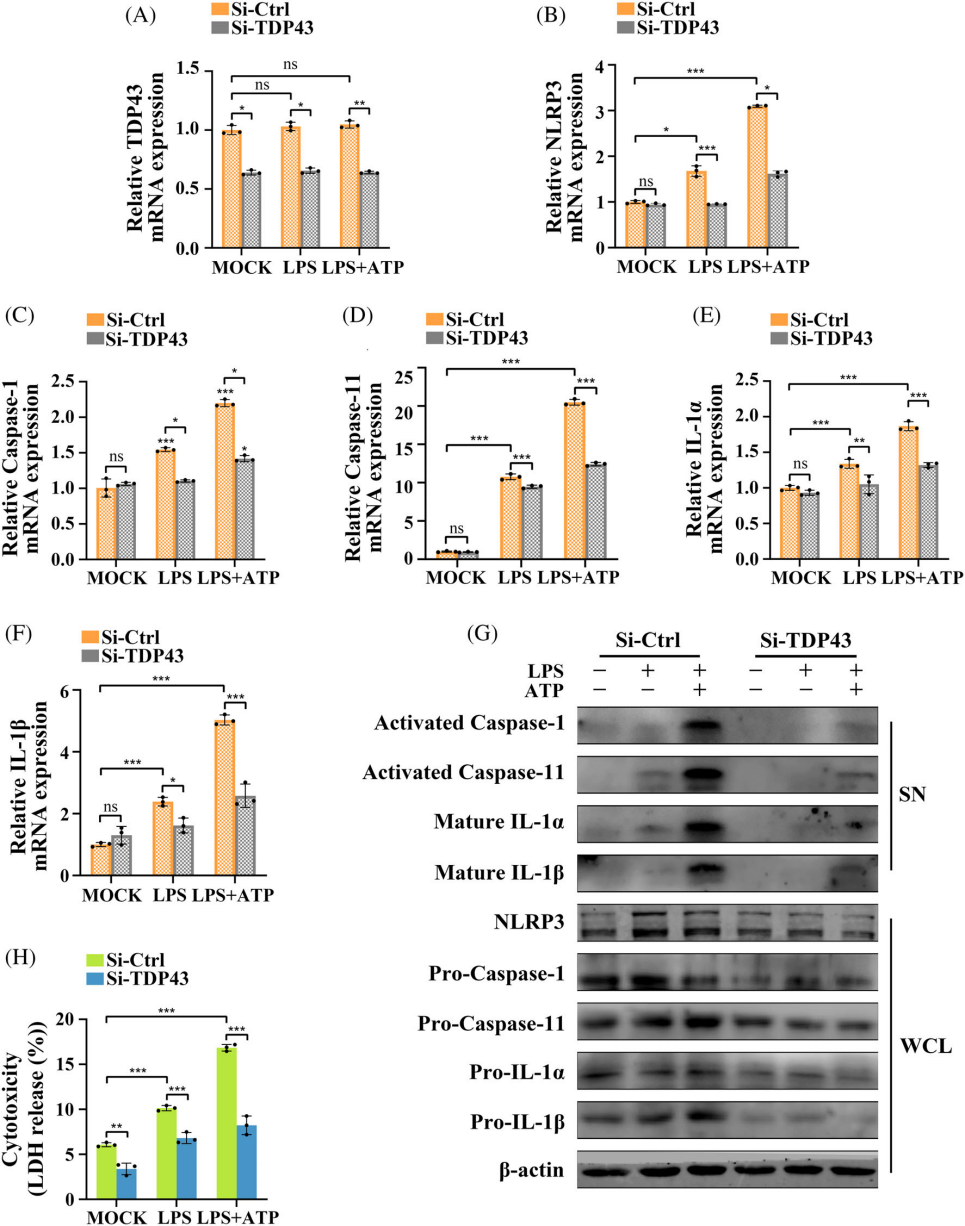
TDP43 Knockdown suppressed the production and activation of caspase‐11 and NLRP3 inflammasome components. (A–H) MGFs from KO mice were transfected with siRNAs (si‐Ctrl and si‐TDP43) and then untreated, treated with LPS alone or treated with LPS + ATP. (A–F) mRNA expressions of TDP43, NLRP3, caspase‐1, caspase‐11, IL‐1α and IL‐1β (RT‐qPCR; *n* = 3). Endogenous control: β‐actin. (G) Protein expressions of indicated molecules were detected by western blotting. Loading control: β‐actin. The experiments were repeated three times. (H) Cytotoxicity was assessed by LDH release as a percentage (%) of total LDH release (*n* = 3). (A‐F, H) Data are presented as mean ± SD. *p < 0.05; **p < 0.01; ***p < 0.001. LPS + ATP, treated with LPS and ATP; LPS, treated with LPS alone; MOCK, untreated without any stimulation; ns, no significance; Si‐Ctrl, MGFs transfected with control siRNAs; Si‐TDP43, MGFs transfected with siRNAs targeting mouse TDP43; SN, supernatant; WCL, whole cell lysate.

TDP43 was knocked down in KO MGFs with a siRNA to investigate whether lincRNA‐EPS alters the production of inflammasome components by interacting with TDP43. LPS or LPS + ATP did not change TDP43 expression, but TDP43 expression was significantly downregulated by siRNA‐TDP43 (Figure 5A).

TDP43 knockdown had no effect on the mRNAs of inflammasome components in resting MGFs, while the mRNA and protein expressions of NLRP3, pro‐caspase‐1, pro‐caspase‐11, pro‐IL‐1α and pro‐IL‐1β were markedly downregulated after LPS/ATP stimulation. In addition, the levels of the activated caspase‐1, activated caspase‐11, mature IL‐1α and mature IL‐1β proteins also decreased remarkably after TDP43 knockdown (Figure 5B–G). In addition, LDH release was also significantly reduced after TDP43 knockdown (Figure 5H). In conclusion, TDP43 knockdown suppresses the expression of components and activation of caspase‐11/NLRP3 inflammasomes in the absence of lincRNA‐EPS. These results are consistent with those from cells overexpressing lincRNA‐EPS.

### 3.7 LincRNA‐EPS overexpression and TDP43 knockdown repressed NF‐κB activation in LPS‐induced MGFs

**FIGURE 6:**
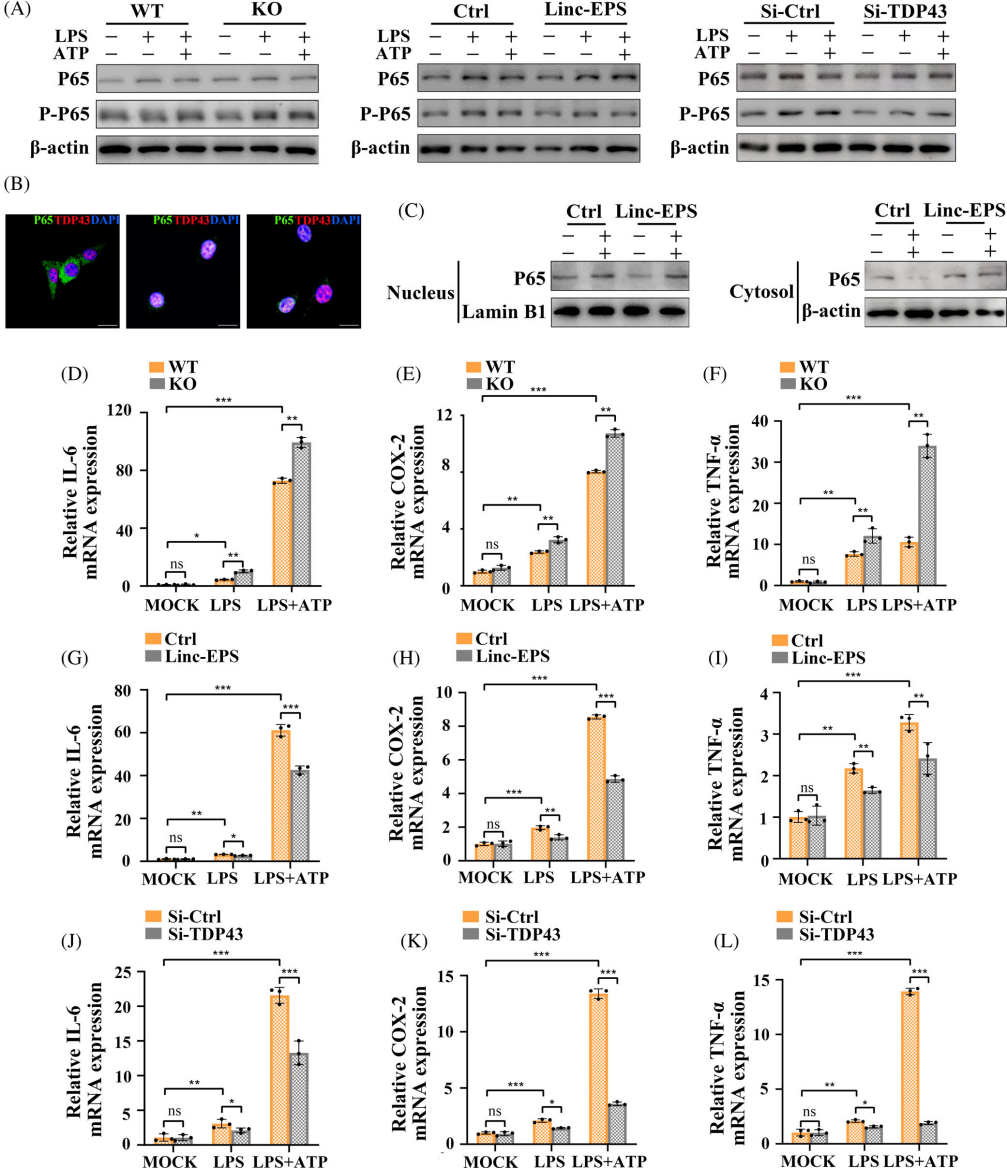
LincRNA‐EPS overexpression and TDP43 knockdown repressed NF‐κB activation in LPS‐induced MGFs. (A) Protein expressions of p65, p‐p65 were detected by western blotting. Loading control: β‐actin. The experiments were repeated three times. (B) Double immunofluorescence labelling IF staining of TDP43 (red) and p65 (green) in untreated, LPS‐ and LPS + ATP‐stimulated MGFs. Nuclei were identified by staining with DAPI (blue), scale bar = 20 μm. (C) P65 expression in the nuclear and cytoplasmic fractions of MGFs with no treatment, LPS stimulation, or LPS + ATP stimulation. The experiments were repeated three times. β‐actin: Cytoplasmic control; Lamin B1: Nuclear control. (D–F) mRNA expressions of IL‐6, COX‐2 and TNF‐α in MGFs from WT and KO mice were untreated, treated with LPS alone or treated with LPS + ATP (RT‐qPCR; *n* = 3). Endogenous control: β‐actin. (G–I) mRNA expressions of mRNA expressions of IL‐6, COX‐2 and TNF‐α in MGFs transfected with control vector and lincRNA‐EPS plasmids, untreated, treated with LPS alone or treated with LPS + ATP (RT‐qPCR; *n* = 3). Endogenous control: β‐actin. (J–L) mRNA expressions of mRNA expressions of IL‐6, COX‐2 and TNF‐α in MGFs transfected with control vector and siRNAs of TDP43, untreated, treated with LPS alone or treated with LPS + ATP (RT‐qPCR; *n* = 3). Endogenous control: β‐actin. (D–L) Data are presented as mean ± SD. *p < 0.05; **p < 0.01; ***p < 0.001. Ctrl, MGFs transfected with negative control vectors; Cytosol, cytoplasmic lysate; KO, MGFs from KO mice; Linc‐EPS, MGFs transfected with lincRNA‐EPS overexpression plasmids; LPS + ATP, treated with LPS and ATP; LPS, treated with LPS alone; MOCK, untreated without any stimulation; ns, no significance; Nucleus, nuclear lysate; Si‐Ctrl, MGFs transfected with control siRNAs; Si‐TDP43, MGFs transfected with siRNAs targeting mouse TDP43; WT, MGFs from WT mice.

TDP43 can bind to the NF‐κB p65 subunit and enhance its phosphorylation and pathway activation in the nucleus of LPS‐induced microglia, where NF‐κB is the upstream transcription factor of inflammasome components.^32^ Hence, to further investigate whether lincRNA‐EPS regulates the expression of inflammasome components by affecting the promotive function of TDP43 in NF‐κB activation, we analysed the effects of lincRNA‐EPS KO, lincRNA‐EPS overexpression and TDP43 knockdown on NF‐κB activation, respectively. The Western blotting results showed a slight increase in RelA mRNA and p65 protein expression under LPS and ATP stimulation, and lincRNA‐EPS KO, lincRNA‐EPS overexpression and TDP43 knockdown had no significant effect on p65 expression. Notably, lincRNA‐EPS KO enhanced the expression of phosphorylated p65 (p‐p65), while lincRNA‐EPS overexpression and TDP43 knockdown reduced p‐p65 expression suggesting the repressed p65 phosphorylation (Figures 6A and S2A–C). Double IF labelling images showed an increase in the nuclear distribution of p65 and co‐localisation with TDP43 in KO MGFs stimulated by LPS and LPS + ATP, indicating TDP43 as the co‐activator of the NF‐κB (Figure 6B). Figures 6C and S2D illustrated that in the LPS + ATP‐treated MGFs in the control group, the ratio of nuclear/cytoplasmic p65 was remarkably increased, while lincRNA‐EPS overexpression significantly reduced it. Subsequent RT‐qPCR assay confirmed the above results. The mRNA levels of NF‐κB downstreaming inflammatory factors (IL‐6, cyclooxygenase‐2 [COX‐2] and TNF‐α^33^) were significantly upregulated after LPS and LPS + ATP stimulation and peaked at LPS + ATP treatment. The mRNA expression of IL‐6, COX‐2 and TNF‐α was significantly elevated in lincRNA‐EPS KO MGFs compared with WT MGFs, whereas the expression of these three mRNAs was markedly suppressed after lincRNA‐EPS overexpression and TDP43 knockdown (Figure 6D–L). Collectively, the above results suggest that TDP43 interacts with p65 and promote NF‐κB activation in the LPS‐induced KO‐MGFs. LincRNA‐EPS overexpression has similar repressive effects with TDP43 knockdown on NF‐κB activation, resulting in the reduced expression of downstream pro‐inflammatory molecules, including inflammasome components.

### 3.8 LincRNA‐EPS rescued pyroptosis in the KO‐MGFs and alleviated periodontal inflammation

**FIGURE 7:**
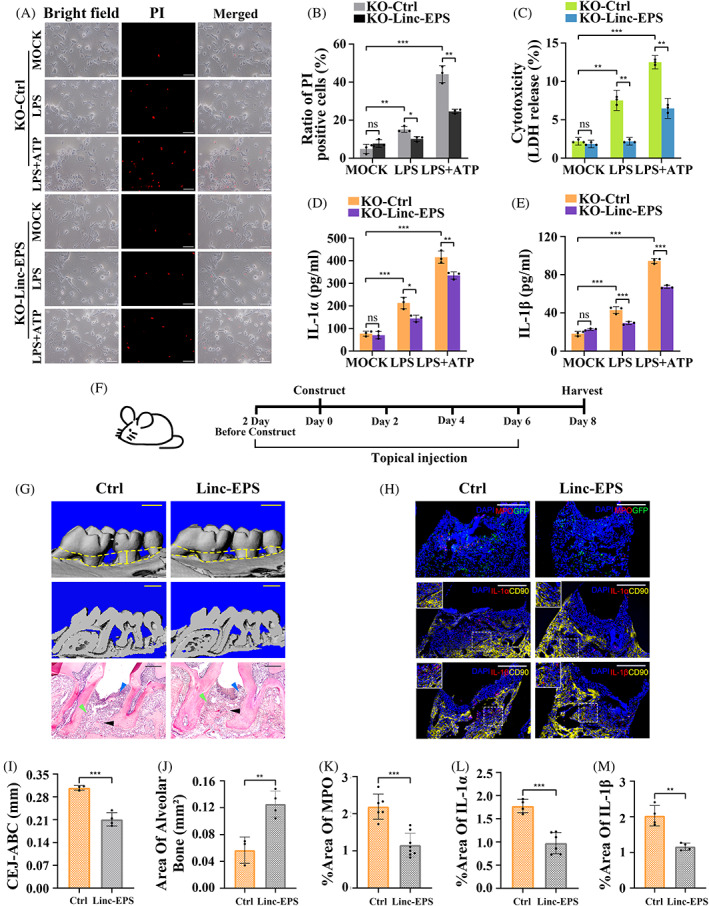
LincRNA‐EPS rescued pyroptosis in the KO MGFs and alleviated periodontal inflammation. (A–E) MGFs from KO mice were transfected with or without lincRNA‐EPS overexpression plasmids and then were untreated, treated with LPS alone or treated with LPS + ATP. (A, B) Cells with or without lincRNA‐EPS overexpression were stained with PI (red), scale bar = 100 μm. The ratio of positive cells was assessed as a percentage (%) of total cells (*n* = 3). Three visual fields were selected for each experiment and the experiments were repeated twice. (C) Cytotoxicity was assessed by LDH release as a percentage (%) of total LDH release (*n* = 3). (D, E) Cell supernatants were detected for mouse IL‐1α and IL‐1β by ELISA (*n* = 3). (F) Timeline of the animal experiment. (G) Reconstructed 3D and 2D micro‐CT images and H&E staining images of the periodontal tissues from Ctrl and linc‐EPS groups, scale bar = 1 mm for micro‐CT images; 200 μm for H&E images. Alveolar bone (black arrow), periodontal ligament (green arrow), inflammatory cell infiltration (blue arrow). (H) Representative IF staining of MPO, IL‐1α, IL‐1β (red) and CD90 (yellow) in the periodontal tissues from Ctrl and linc‐EPS groups, the co‐localisation (orange) of IL‐1α or IL‐1β with CD90 antibodies was magnified in the square box. Cells transfected with lentivirus were identified by green fluorescent protein (green). Nuclei were identified by staining with DAPI (blue), scale bar = 100 μm. (I) The distances (mm) of cementoenamel junction to alveolar bone crest (CEJ‐ABC) of periodontal tissues from respective groups. (J) The area of alveolar bone (mm^2^) of periodontal tissues from respective groups. Percentage of MPO (K), IL‐1α (L), IL‐1β (M) stained area over total area. (B–E, I–M) Data are presented as mean ± SD. *p < 0.05; **p < 0.01; ***p < 0.001. Ctrl, periodontal tissues from lentivirus (control) local injection group; KO‐Ctrl, KO MGFs transfected with negative control plasmids; KO‐linc‐EPS, KO MGFs transfected with lincRNA‐EPS overexpression plasmids; linc‐EPS, lincRNA‐EPS overexpression lentivirus local injection group; LPS + ATP, treated with LPS and ATP; LPS, treated with LPS alone; MOCK, untreated without any stimulation; ns, no significance.

Based on the above findings, lincRNA‐EPS plasmids were transfected into KO MGFs to rescue its function, aiming to confirm its negative effects on pyroptosis. LDH assay and PI staining revealed that, upon stimulation, the regaining of lincRNA‐EPS decreased the pyroptosis in KO MGFs (Figure 7A–C). ELISA also showed that the substantial increase in IL‐1α and IL‐1β secretion was also significantly reduced after lincRNA‐EPS restoration (Figure 7D,E). Subsequently, we applied the lincRNA‐EPS overexpression lentiviral vectors topically to the periodontium of the maxillary second molar of WT mice with periodontitis (Figure 7F) and observed the expression of lincRNA‐EPS marked by a green fluorescent protein. As expected, CT and H&E results indicated that compared with the control vector‐treated group, mice in the lincRNA‐EPS overexpression‐treated group had significantly decreased CEJ‐ABC distance, area of alveolar bone loss, as well as reduced inflammatory cell infiltration and fibrous destruction, while MPO expression and aggregation, IL‐1α and IL‐1β expression were significantly reduced (Figure 7G–M). Thus, restoration of lincRNA‐EPS expression rescued IL‐1α/1β‐dependent pyroptosis in MGFs, and its topical application attenuated periodontal inflammation.

## 4 DISCUSSION

In this study, we found that lincRNA‐EPS could inhibit the expression of caspase‐11/NLRP3 inflammasome components in the LPS‐induced MGFs by compromising the activation of NF‐κB signalling pathway via interacting with TDP43. In vivo results showed that lincRNA‐EPS is involved in the pathogenesis of periodontitis, its local interventions effectively alleviated the inflammation.

In terms of caspase‐11/NLRP3 inflammasomes, LPS first activates toll‐like receptor 4 (TLR4)/NF‐κB signalling to promote the transcription of their components. Subsequently, NLRP3 inflammasome is assembled and pro‐caspase‐1 is activated after ATP, K+ efflux or other stimulation, resulting in gasdermin D cleavage along with IL‐1β secretion.^8^, ^34^ In contrast, caspase‐11 directly binds to intracellular LPS, causing caspase‐11 activation, followed by ATP release and P2X7 ATP‐gated ion channel activation and mature IL‐1α/1β secretion.^8^, ^35^, ^36^ Evidence has suggested that K^+^ efflux and ATP release after caspase‐11 activation further activate NLRP3 inflammasome, ultimately resulting in pyroptosis.^8^, ^37^ We found that LPS upregulated the transcription and expression of each component, namely, NLRP3, caspase‐1, caspase‐11, IL‐1α and IL‐1β. More importantly, the activated caspase‐11 and mature IL‐1α were detectable in each control group after LPS treatment, while the levels of these four active products significantly increased after LPS + ATP treatment. Based on these findings, LPS primarily activates caspase‐11, but LPS together with ATP activates both caspase‐11 and NLRP3 inflammasomes in the MGFs. Interestingly, a further increase was noted in the mRNA expression of each molecule in cells treated with LPS + ATP as compared to cells treated with LPS alone. One possible explanation is that ATP increases the transcription of inflammasome components through the P2X7/NF‐κB pathway, consistent with the results reported by Li et al.^38^, ^39^, ^40^

In periodontitis, most of the currently reported lncRNAs can competitively bind to miRNAs in the cytoplasm to regulate their downstream effectors in modulating inflammation.^41^ For instance, in the LPS‐induced human GFs, the lncRNA MALAT1 sponges miR‐20a to activate TLR4 signalling and then promote IL‐6 and IL‐8 expression.^42^ We confirmed that lincRNA‐EPS was located primarily in the nucleus of MGFs in both resting and inflammatory contexts. Nuclear lncRNAs can regulate the expression of inflammatory genes primarily via: (1) binding to RNA‐binding proteins (such as hnRNPs),^20^, ^43^, ^44^ (2) interacting with transcription or splicing factors (such as NF‐κB)^18^, ^45^ or (3) affecting epigenetic regulation.^18^, ^46^ In the present study, lincRNA‐EPS reduced the gene expression and protein activation of the inflammasome components in the MGFs after LPS or LPS + ATP treatment. Although the expression of pro‐proteins and genes may not fully correspond (Figure 3G), the difference did not negate the transcriptionally repressive effect on the expression of these components. Unlike the findings reported by Atianand et al.,^20^ our results showed that lincRNA‐EPS interacted with TDP43 instead of hnRNPL in the MGFs and restricted the production and activation of caspase‐11/NLRP3 inflammasome components, while hnRNPL in macrophages mainly affects ASC expression and NLRP3 inflammasome activation. This difference may be attributed to the use of distinct cell types and/or different treatments.

TDP43 binds to RNA or DNA to regulate RNA‐related biological processes, which are closely related to the inflammatory signalling pathways.^47^, ^48^ Moreover, TDP43 has been confirmed to upregulate the expression of NLRP3 and IL‐1β and activate the NLRP3 inflammasome in the microglia through the NF‐κB pathway.^25^, ^48^ Our results showed that in the KO MGFs, TDP43 knockdown reduced the expression of caspase‐11/NLRP3 inflammasome components under LPS stimulation, in turn, supports the pro‐inflammatory activity of TDP43, and this consequence in the transcriptional repression of inflammasome components is consistent with the effect of lincRNA‐EPS overexpression. We found that TDP43 co‐localised with p65 in the nucleus of KO MGFs under LPS stimulation, but it co‐localised with lincRNA‐EPS in the nucleus of the LPS‐treated WT MGFs. Nuclear TDP43 has been reported to directly interact with NF‐κB p65 subunit and function as a co‐activator of NF‐κB to promote the expression of inflammatory factors after LPS treatment.^31^, ^49^, ^50^ As NF‐κB is an upstream signal of LPS‐induced transcription of inflammasome components, we speculate that the lincRNA‐EPS/TDP43 axis may affect the downstream inflammasome components by restricting the co‐activating function of TDP43 to NF‐κB. The subsequent results demonstrated that lincRNA‐EPS overexpression and TDP43 knockdown repressed NF‐κB activation and transcription of its downstream inflammatory factors. Therefore, it is highly likely that the overexpressed lincRNA‐EPS competitively reduced the amount of TDP43 that co‐activated with NF‐κB by interacting with TDP43 to consequently exert a suppressive effect on the transcription of caspase‐11/NLRP3 inflammasome components. However, this suppressive effect of the lincRNA‐EPS/TDP43 axis on the expression of inflammasomes via restricting NF‐κB activation is still an indirect mechanism, the more direct and central mechanism should be explored in further studies.

GFs in gingival tissues are capable of responding to pathogens and inflammatory signals, secreting inflammatory cytokines including IL‐6 and IL‐8. They recruit neutrophils and lymphocytes into inflamed gingival tissues and interact with each other by producing chemokines such as C‐X‐C motif chemokine ligand 1 (CXCL1) to co‐modulate host inflammatory response and immune homeostasis.^51^ Furthermore, GFs can also secret IL‐1β and IL‐18 via inflammasome activation when triggered by pathogens.^4^, ^13^ Not only that, GFs are also involved in regulating alveolar bone resorption and connective tissue destruction. *Porphyromonas gingivalis*‐induced GFs can promote osteoclast production. A recent study found that the secretion of early active cathepsin K (Ctsk) in GFs leads to connective tissue degradation and periodontal pocket formation.^52^ Thus, the investigation of MGFs in this study is of great significance in exploring the pathological mechanisms of periodontal inflammation. According to IF staining in Figures 1F and 7H, MGFs (CD90+) from the periodontitis group, were observed with higher expression of IL‐α/β, in which group, more severe bone resorption, periodontal ligament destruction and inflammatory cell infiltration occurred, suggesting that the inflammasome activation in GFs is partially involved in periodontal inflammation. However, inflammatory cells still play a central role in periodontitis.^3^ The ability of lincRNA‐EPS to inhibit the expression of inflammatory genes in macrophages and reduce tissue inflammation has been confirmed earlier, so our results exhibiting the negative regulatory function of lincRNA‐EPS on periodontal inflammation may contribute a complementary to and an extension of this repressing ability with respect to stromal cells in the process of periodontitis. Whereas further trails are needed for the specific regulation mechanisms among cells in periodontal inflammation.

**FIGURE 8:**
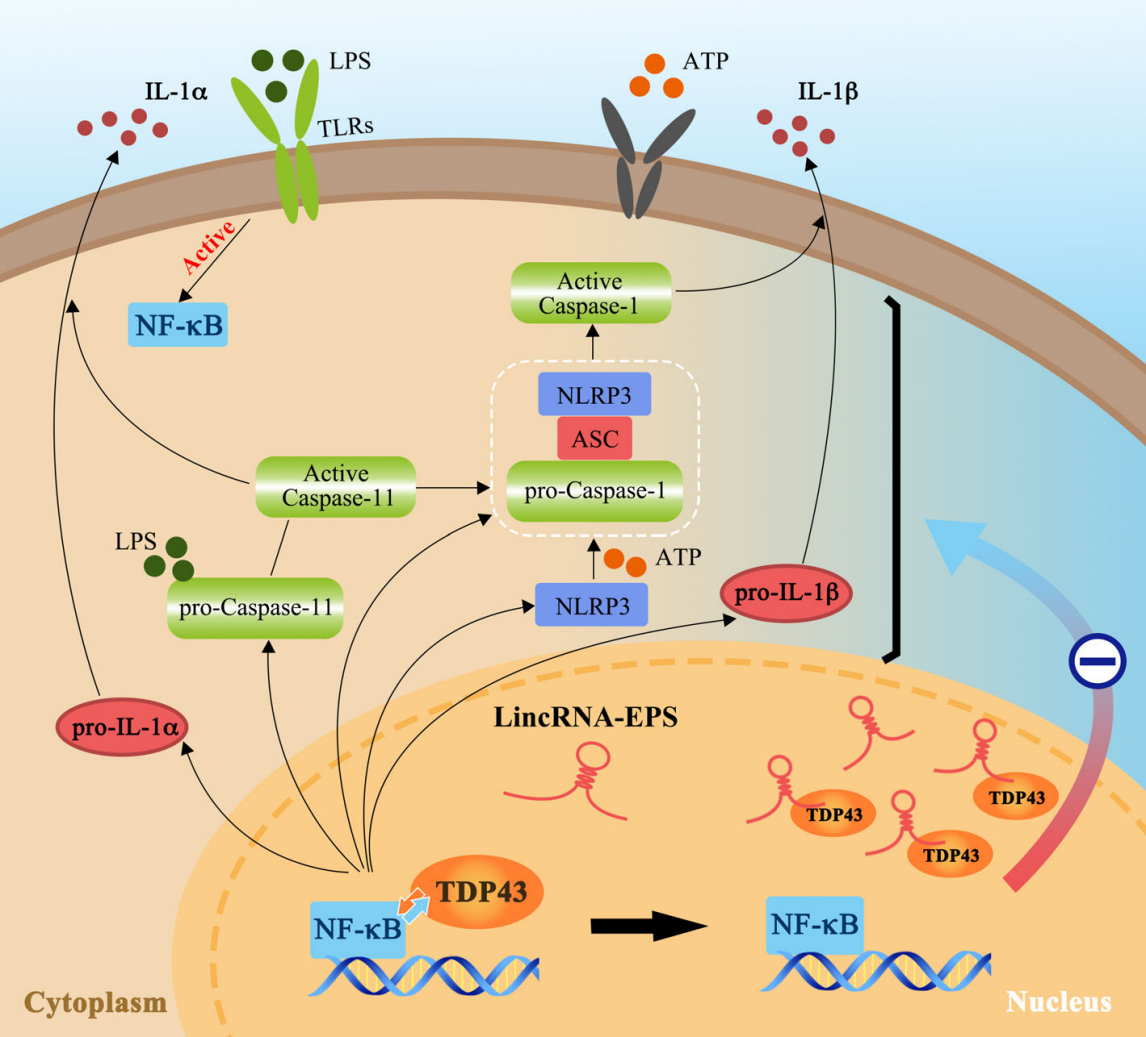
The proposed schematic diagram. In the LPS‐induced inflammatory environment, TDP43, as a co‐activator of NF‐κB, can activate caspase‐11 and NLRP3 pathways. Whereas the overexpressed lincRNA‐EPS may competitively reduce the amount of TDP43 co‐activating with p65 to compromise NF‐κB activation and the transcriptional expression of caspase‐11 and NLRP3 inflammasome components (NLRP3, caspase‐1, caspase‐11, IL‐1α and IL‐1β), subsequently inhibit the activation of these inflammasomes.

In conclusion, the present study reveals that lincRNA‐EPS topical overexpression relieves periodontal inflammation. Mechanically, lincRNA‐EPS overexpressed in the LPS‐treated MGFs interacts with TDP43, restricting its promotive function in NF‐κB activation and then inhibiting the expression of caspase‐11/NLRP3 inflammasome components, which impairs their activation and attenuates pyroptosis in the MGFs (Figure 8). Understanding the negative effect of lincRNA‐EPS on the inflammasomes in MGFs and periodontal inflammation may be helpful for the control of the progression of periodontitis and for the development of therapeutic strategies for other inflammasome‐related diseases.

## AUTHOR CONTRIBUTIONS

Jiansheng Su conceived and designed the project. Anni Hu prepared the materials, conducted all the in vitro experiments, performed all the data analysis and drew the schematic figures. Fan Xiao and Wenjing Wu performed the animal experiments and the data acquisition. Huilin Xu participated in collecting the gingiva tissues. All authors discussed and interpreted the results. Anni Hu completed the draft and Jiansheng Su revised the main manuscript. All authors modified and gave final approval of this work.

## CONFLICT OF INTEREST STATEMENT

The authors declare no conflict of interest.

## Supporting information


**FIGURE S1.** (A–C) The relative expression of NLRP3, IL‐1α, IL‐1β proteins were evaluated by grey value analysis of western blotting images corresponding Figure 2F. Data are presented as mean ± SD. *p < 0.05; **p < 0.01; ***p < 0.001. ns, no significance. MOCK, untreated without any stimulation; LPS, treated with LPS alone; LPS + ATP, treated with LPS and ATP.

**FIGURE S2.** (A–C) mRNA expressions of RelA in MGFs from indicated groups (RT‐qPCR; *n* = 3). Endogenous control: β‐actin. (D) The ratio of nuclear/cytoplasmic p65 in MGFs from indicated groups according to the result in Figure 6C. Data are presented as mean ± SD. *p < 0.05; **p < 0.01; ***p < 0.001. ns, no significance. MOCK, untreated without any stimulation; LPS, treated with LPS alone; LPS + ATP, treated with LPS and ATP; WT, MGFs from WT mice; KO, MGFs from KO mice. Ctrl, MGFs transfected with negative control vectors; Linc‐EPS, MGFs transfected with lincRNA‐EPS overexpression plasmids. Si‐Ctrl, MGFs transfected with control siRNAs; Si‐TDP43, MGFs transfected with siRNAs targeting mouse TDP43. NC‐0, MGFs transfected with negative control vectors without stimulation; NC‐L + A, MGFs transfected with negative control vectors under LPS + ATP stimulation; Linc‐EPS‐0, MGFs transfected with lincRNA‐EPS overexpression plasmids without stimulation; Linc‐EPS‐L + A, MGFs transfected with lincRNA‐EPS overexpression plasmids with LPS + ATP stimulation.

**TABLE S1.** siRNAs for TDP43.
**TABLE S2.** Primers for DNA templates.

## Data Availability

The data that support the findings of this study are available from the corresponding author.
