# Supplementary figures and images for: LincRNA‐EPS inhibits caspase‐11 and NLRP3 inflammasomes in gingival fibroblasts to alleviate periodontal inflammation
Source: Cell Prolif. 2023 Sep 14;57(1):e13539. doi: 10.1111/cpr.13539 (PMC10771112; doi:10.1111/cpr.13539)

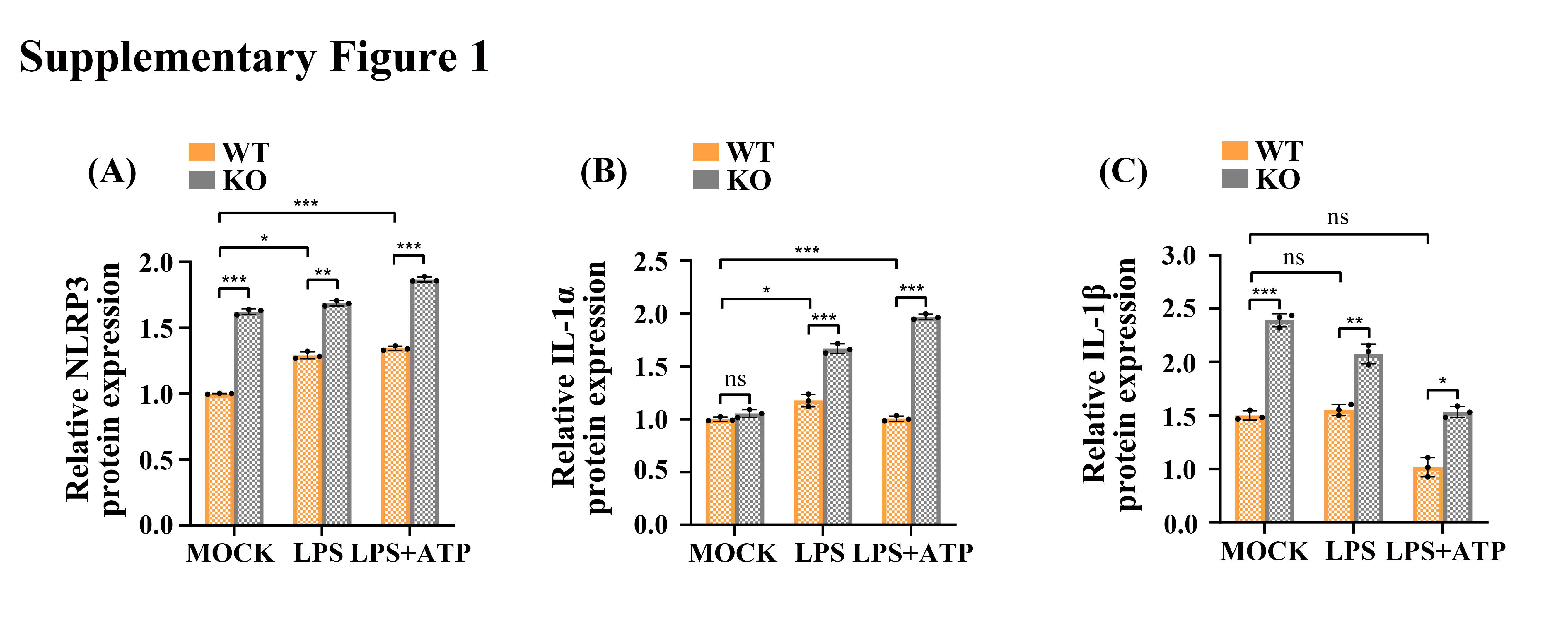

Supplement: Supplementary file 1 — FIGURE S1. (A–C) The relative expression of NLRP3, IL‐1α, IL‐1β proteins were evaluated by grey value analysis of western blotting images corresponding Figure 2F. Data are presented as mean ± SD. *p < 0.05; **p < 0.01; ***p < 0.001. ns, no significance. MOCK, untreated without any stimulation; LPS, treated with LPS alone; LPS + ATP, treated with LPS and ATP. [file CPR-57-e13539-s002.tif]

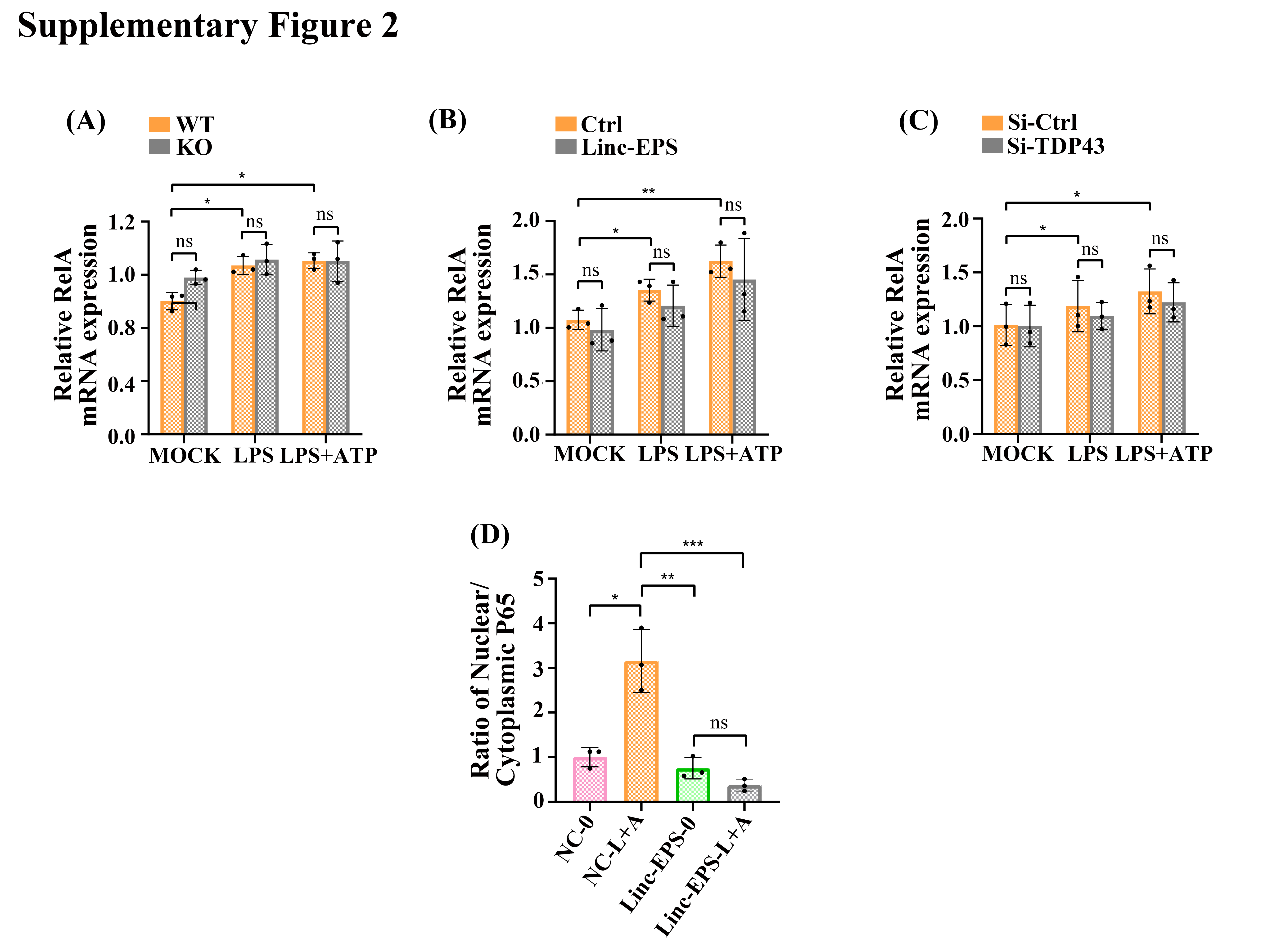

Supplement: Supplementary file 2 — FIGURE S2. (A–C) mRNA expressions of RelA in MGFs from indicated groups (RT‐qPCR; n = 3). Endogenous control: β‐actin. (D) The ratio of nuclear/cytoplasmic p65 in MGFs from indicated groups according to the result in Figure 6C. Data are presented as mean ± SD. *p < 0.05; **p < 0.01; ***p < 0.001. ns, no significance. MOCK, untreated without any stimulation; LPS, treated with LPS alone; LPS + ATP, treated with LPS and ATP; WT, MGFs from WT mice; KO, MGFs from KO mice. Ctrl, MGFs transfected with negative control vectors; Linc‐EPS, MGFs transfected with lincRNA‐EPS overexpression plasmids. Si‐Ctrl, MGFs transfected with control siRNAs; Si‐TDP43, MGFs transfected with siRNAs targeting mouse TDP43. NC‐0, MGFs transfected with negative control vectors without stimulation; NC‐L + A, MGFs transfected with negative control vectors under LPS + ATP stimulation; Linc‐EPS‐0, MGFs transfected with lincRNA‐EPS overexpression plasmids without stimulation; Linc‐EPS‐L + A, MGFs transfected with lincRNA‐EPS overexpression plasmids with LPS + ATP stimulation. [file CPR-57-e13539-s003.tif]
