# Supplementary material for: LincRNA‐EPS inhibits caspase‐11 and NLRP3 inflammasomes in gingival fibroblasts to alleviate periodontal inflammation
Source: Cell Prolif. 2023 Sep 14;57(1):e13539. doi: 10.1111/cpr.13539 (PMC10771112; doi:10.1111/cpr.13539)
Supplement: Supplementary file 3 — TABLE S1. siRNAs for TDP43. TABLE S2. Primers for DNA templates. [file CPR-57-e13539-s001.docx]

**Supplementary Materials**

**LincRNA-EPS inhibits caspase-11 and NLRP3 inflammasomes**

**in gingival fibroblasts to alleviate periodontal inflammation**

Anni Hu, Fan Xiao, Wenjing Wu, Huilin Xu, Jiansheng Su

**1. Methods and Materials**

**1.1 Antibodies**

The primary antibodies used for western blotting, RNA immunoprecipitation (RIP) and immunofluorescence (IF) staining included anti-NLRP3 (#bs-10021R, 1:1000; Bioss, Beijing, China), anti-caspase-1 (#NBP1-45433SS, 1:1000; Novus Biologicals, Littleton, CO, USA), anti-caspase-11 (#ab180673, 1:1000; Abcam, Cambridge, UK), anti-IL-1α (#bs-4947R, 1:1000; Bioss), anti-IL-1β (#NB-600633, 1:1000; Novus), anti-TDP43 (#3448, 1:1000, 1:100, 1:50; Cell Signaling Technology, Danvers, MA, USA), anti-IgG (1:50; Merck Millipore, Billerica, MA, USA), anti-MPO (#AF3667, 1:100; R&D System, Minneapolis, USA), anti-CD90 (#sc-53116, 1:100; Santa Cruz Biotechnology, Santa Cruz, CA, USA), anti-GAPDH (#AF7021, 1:1000; Affinity Biosciences, Zhenjiang, China ), anti-Lamin B1 (#ab133741,1:1000; Abcam) and anti-β-actin (#AF7018, 1:1000; Affinity), anti-p65 (#6956, 1:1000; Cell Signaling Technology), anti-p-p65 (#AF2006, 1:1000; Affinity), anti-p65 (#AF5006, 1:1000; Affinity) antibodies. The secondary antibodies used for Western blotting and immunofluorescence staining included goat anti-rabbit IgG (H+L) (#5366, 680 Conjugate, 1:10,000; Cell Signaling Technology), goat anti-rabbit IgG (H+L) (#S0008, FITC Conjugate, 1:200; Affinity), goat anti-mouse IgG (H+L) (#S0005, 594 Conjugate, 1:200; Affinity), and HRP-labeled goat anti-rabbit IgG (H+L) (#A0208, 1:1000; Beyotime, Shanghai, China).

**1.2 Micro-CT analysis**

Fixed mouse maxillae were scanned using a micro-CT system (UCT50, ScancoMedical AG, Switzerland) at the scanning resolution of 10 μm. Reconstructed thress-dimensional (3D) microstructural images and 2D images were obtained by the affiliated analyzing software. The distances between the ementoenamel junction (CEJ) and the alveolar bone crest (ABC) from the mesial, middle and distal sides of the upper second molar, were measured using the Image J software, average values were taken for analysis. The area of alveolar bone was also measured using the Image J software. The sum the mesial, distal and furcation area were taken for next analysis.

**1.3 Cell culture**

Primary mouse gingival fibroblasts (MGFs) were obtained as previously reported: gingival tissues were harvested from 4-week-old male C57BL/6J mice and minced with scissors. The tissue blocks were cultured in complete DMEM supplemented with 1% penicillin–streptomycin solution (Gibco) and 10% fetal bovine serum (FBS; Gibco, Grand Island, NY, USA) in flasks at 37℃ with 5% CO_2_ for 2 weeks before the first passaging^30^. MGFs of the third to sixth passages were used for following experiments. The NIH3T3 cell line (American Type Cell Culture, ATCC; VA, USA) was also cultured under the same conditions.

**1.4 PI staining**

MGFs were cultured in 24-well plates and treated as indicated. Propidium iodide (1 µg/mL, Sigma) was added to the medium. The cells were incubated at 4℃ for 30 min away from light. Images of pyroptotic cells were captured using a fluorescence microscope (Nikon).

**1.5 Transmission electron microscope (TEM)**

LPS+ATP induced MGFs and control MGFs were fixed by 2.5% glutaraldehyde were scraped into 1.5ml centrifuge tubes then stored at 4 °C. After washing and refixing with osmium tetroxide solution, the samples were made into nanoscale slices, stained by uranium acetate and lead citrate. The samples were observed by TEM (Hitachi Ltd., Tokyo, Japan).

**1.6 Protein extraction and western blotting**

Proteins from the cell culture supernatant were extracted by centrifuging the sample at 4000 g in a 3KD ultrafiltration tube (Merck Millipore, Bedford, MA, USA). Cellular proteins were obtained by lysing cells with RIPA lysis buffer (Beyotime). Proteins were quantified, mixed with loading buffer (Beyotime), electrophoretically separated on SDS–PAGE gels (Beyotime) and then transferred to nitrocellulose (NC) membranes (BBI), blocked with 5% BSA (Beyotime). After overnight incubation at 4°C with the primary antibodies, the NC membrane was incubated with the secondary antibody for 1 hour at room temperature. The proteins were visualized using Amersham ImageQuant 800 imaging systems (Cytiva, Tokyo, Japan) and Odyssey (LI-COR, Lincoln, NE, USA).

**1.7 ELISA**

MGFs were preseeded and treated in 6-well plates. The cell culture supernatant was collected and assayed with ELISA Kits (MEIMIAN, Yancheng China) as directed by the manufacturer.

**1.8 LDH assay**

MGFs were preseeded and treated in 6-well plates. LDH released into the cell culture supernatant was measured using the LDH Cytotoxicity Assay Kit (Beyotime) according to the manufacturer’s protocol. The percentage of LDH released was calculated as 100 × (experimental LDH level − spontaneous LDH level) / (maximum LDH level − spontaneous LDH level).

**1.9 Cytosolic and nuclear fractionation and RNA/protein isolation**

MGFs were collected on ice for nuclear and cytoplasmic RNA/protein extraction. Then, the PARIS Kit (Invitrogen, Carlsbad, CA, USA) was used to obtain the cytoplasmic and nuclear samples in accordance with the manufacturer’s instructions. Briefly, cell fractionation buffer was added to cells, the mixture was centrifuged for 5 min and the supernatant was collected as cytoplasmic sample. Then, cell disruption buffer was added to the sediment to obtain nuclear sample. These samples were directly used for subsequent RNA purification and RT-qPCR or western blotting.

**2.0 LincRNA-EPS lentiviral expression vector construction and topical injection**

Green fluorescent protein (GFP)-labeled lincRNA-EPS-overexpressing and control lentiviral vectors were prepared by RiboBio. The lentiviral vectors were injected into mice maxillary right second molar at mid-buccal side, mesial-palatal side, mid-palatal side, and distal-palatal injection sites with 5ul for each site, with a virus titer of 2 × 108TU/ml every other day, starting 2 d before the construction of the periodontitis model, totally for 5 times.

**2. siRNAs for TDP43 Knockdown**

**Table 1 siRNAs for TDP43**

| siRNA name | Target sequence |
| --- | --- |
| si-m-TDP43_001 | TCAACTATCCCAAAGATAA |
| si-m-TDP43_002 | CACAACGACATATGATAGA |
| si-m-TDP43_003 | GAGCCTTTGAGAAGCAGAA |

The most effective siRNA was selected for subsequent experiments.

**3. Primers for DNA templates in Pull-down assay**

**Table 2 Primers for DNA templates**

| Primer name | Sequence |
| --- | --- |
| LincRNA-EPS-  sense-Forward  LincRNA-EPS-  sense-Reverse | TAATACGACTCACTATAGGGATTGGCCCTGAGCTCCAGGATGTCA  TAAATCATAAGGAAATTTATTTTAA |
| LincRNA-EPS-  antisense-Forward  LincRNA-EPS-  antisense-Reverse | ATTGGCCCTGAGCTCCAGGATGTCA  TAATACGACTCACTATAGGGTAAATCATAAGGAAATTTATTTTAA |

**Figure Legends**

**Supplementary Figure 1**

**(A-C)** The relative expression of NLRP3, IL-1α, IL-1β proteins were evaluated by gray value analysis of western blotting images corresponding Fiigure 2F. data are presented as mean ± SD. **P* < 0.05, ***P* < 0.01, ****P* < 0.001. **ns**, no significance. **MOCK,** untreated without any stimulation; **LPS**, treated with LPS alone; **LPS+ATP**, treated with LPS and ATP.

**Supplementary Figure 2**

**(A-C)** mRNA expressions of RelA in MGFs from indicated groups, (RT-qPCR; n=3). Endogenous control: β-Actin. **(D) The ratio of nuclear/cytoplasmic p65 in MGFs** from indicated groups according to the result in Figure 6C. Data are presented as mean ± SD. **P* < 0.05, ***P* < 0.01, ****P* < 0.001. **ns**, no significance. **MOCK,** untreated without any stimulation; **LPS**, treated with LPS alone; **LPS+ATP**, treated with LPS and ATP; **WT**, MGFs from WT mice; **KO**, MGFs from KO mice. **Ctrl**, MGFs transfected with negative control vectors; **Linc-EPS**, MGFs transfected with lincRNA-EPS overexpression plasmids. **Si-Ctrl**, MGFs transfected with control siRNAs; **Si-TDP43**, MGFs transfected with siRNAs targeting mouse TDP43. **NC-0,** MGFs transfected with negative control vectors without stimulation; **NC-L+A,** MGFs transfected with negative control vectors under LPS+ATP stimulation; **Linc-EPS-0,** MGFs transfected with lincRNA-EPS overexpression plasmids without stimulation; **Linc-EPS-L+A,** MGFs transfected with lincRNA-EPS overexpression plasmids with LPS+ATP stimulation.
